# A case of unprovoked, precarious, patent foramen ovale protuberance

**DOI:** 10.1530/ERP-17-0027

**Published:** 2017-07-10

**Authors:** H Z R McConkey, M Ghosh-Dastidar, S R Redwood, V Bapat

**Affiliations:** The King’s College London British Heart Foundation Centre of Excellence, The Rayne Institute, London, UK

## Abstract

This is a case of a precarious thrombotic mass straddling a patent foramen ovale which had already embolised to the pulmonary circulation. The diagnosis was initially deceptive and management challenging.

**Learning points:**

- Echocardiography is mandated and can change management in haemodynamically unstable patients with pulmonary emboli.
- Pulmonary embolism can be life-threatening.
- The authors propose that urgent cardiac surgery is the safest treatment in the setting of highly mobile, large volume, intra-cardiac thrombus.

## Background

This case outlines the challenges in diagnosing pulmonary emboli, and how instrumental the role of echocardiography can be. The authors advocate urgent cardiac surgery as the safest treatment.

## Case presentation

We report the case of a 66-year-old male who attended the emergency department with back pain. An unenhanced renal tract computed tomography (CT) scan demonstrated right lung basal atelectasis with no renal tract calcification, and the patient was discharged home with naproxen and co-amoxiclav in view of raised inflammatory markers (WCC 15.4 × 10^9^, CRP 94 mg/L, eGFR 49 mL/min/1.7 m^2^, Creatinine 128 mmol/L). 24 h later, the patient attended another hospital with a history of sudden onset dyspnoea whilst gardening two days prior, and right chest/flank pain made worse by inspiration. The patient was hypoxic, diaphoretic and in discomfort, prompting admission. Arterial blood gas sampling revealed type 1 respiratory failure on FiO2 24% with a compensated respiratory alkalosis (pH 7.4, pCO_2_ 3.8, pO_2_ 8.6, Lactate 0.9, BE −5.4, HCO_3_ 20.5). Blood pressure was 95/68 mmHg, heart rate 98 beats per minute and electrocardiography showed inverted T waves in leads V1–4 with right axis deviation. There was a further rise in WCC and CRP to 16.6 and 235, respectively, however chest X ray revealed no frank consolidation or pulmonary oedema.

The patient’s background history consisted of a cholecystectomy in 2009 for cholelithiasis, hiatus hernia, and possible hypertension, and had never smoked. Current medications only comprised of bisoprolol 1.25 mg od and co-codamol prn.

## Investigation

**Figure 1:**
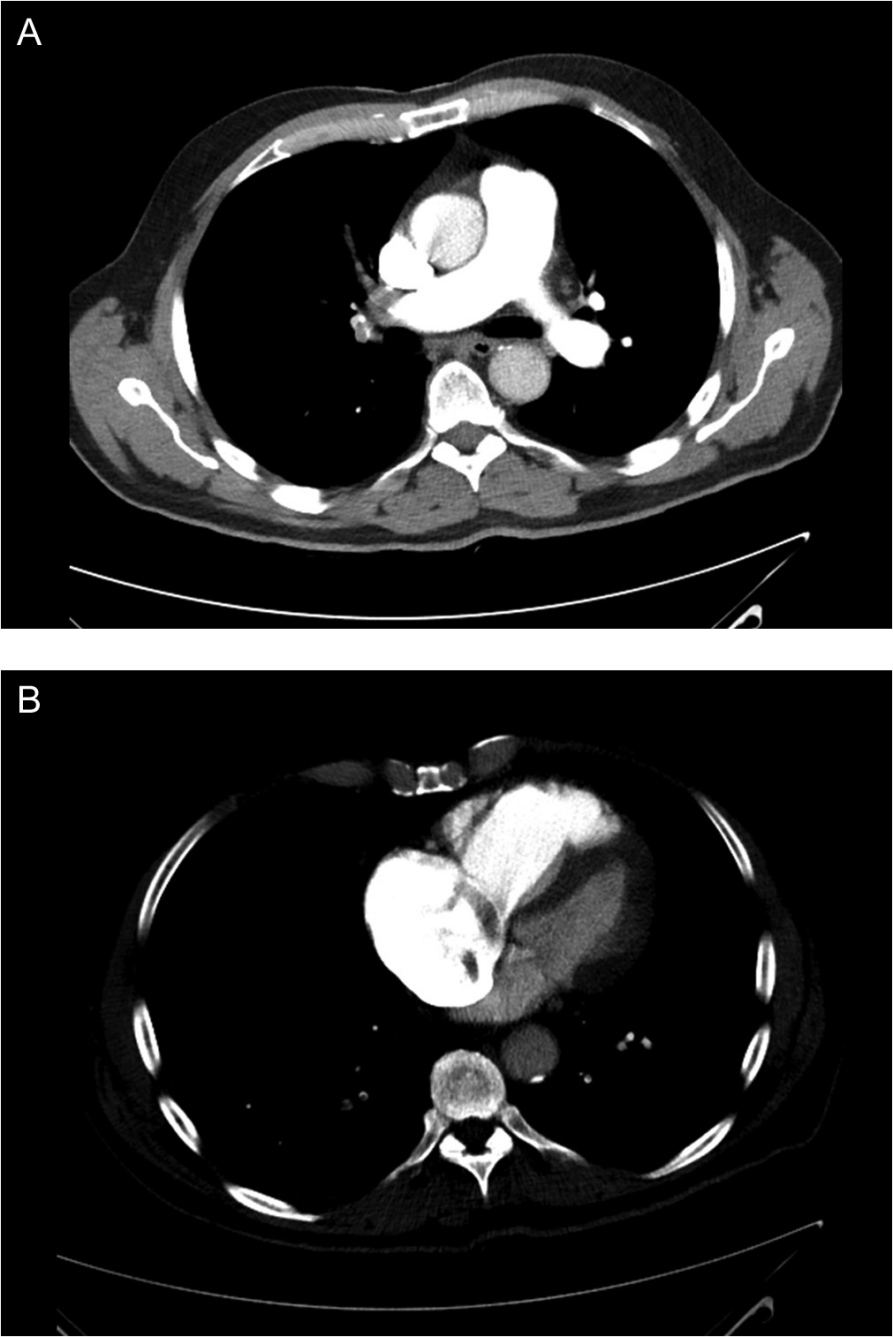
(A) CT pulmonary angiogram demonstrating emboli in the right pulmonary arteries with dilatation of the main pulmonary trunk. (B) CT pulmonary angiogram demonstrating intra-cardiac filling defects.

The patient was diagnosed with a probable pulmonary embolism and commenced on treatment dose subcuta­neous low molecular weight heparin and intravenous antibiotics. High sensitivity troponin T was elevated at 49 ng/L. A CT pulmonary angiogram confirmed extensive, bilateral pulmonary emboli (Figs 1A and B) involving most of the main branches of the pulmonary arteries, with dilatation of the main pulmonary trunk (35 mm), reflux of contrast into the intrahepatic inferior vena cava and intraventricular septal wall flattening in keeping with right heart strain. There was also note of a non-specific sub-pleural nodule measuring up to 1.6 cm within the lingula.

**Figure 2:**
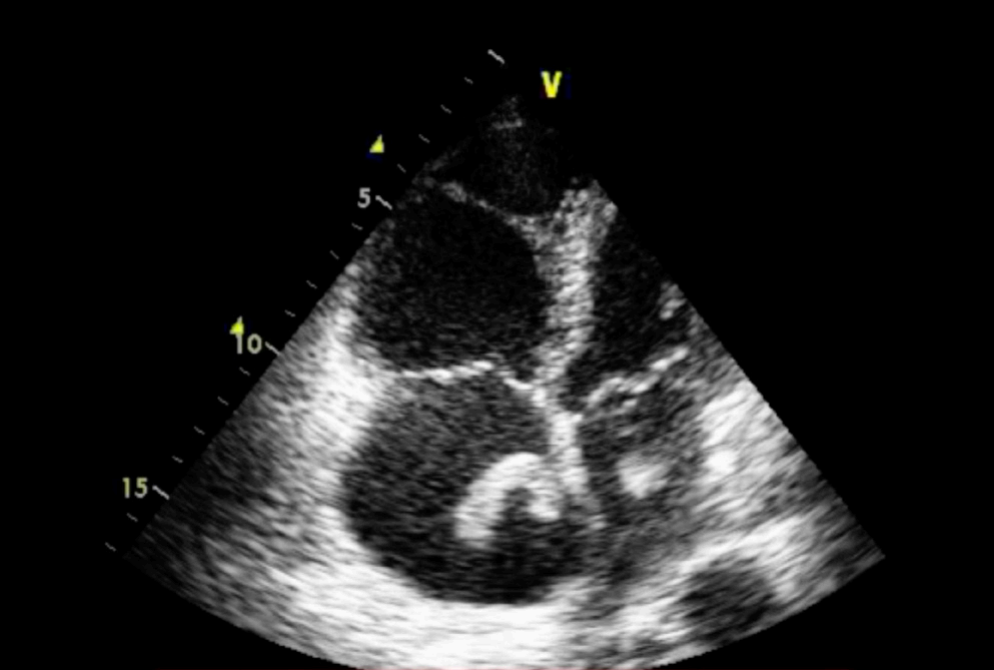
Transthoracic echocardiogram demonstrating right heart dilatation and bi-atrial masses

An echocardiogram the following morning revealed large, highly mobile masses in both atria (Fig. 2 and Videos 1 and 2) which were seen to protrude through the mitral and tricuspid valves in diastole. The right heart was dilated and systolic function impaired (TAPSE 7mm), and there was associated severe pulmonary hypertension (TR V_max_ 52 mmHg and a dilated inferior vena cava, minimally responsive to inspiration) despite normal right ventricular (RV) free wall thickness suggesting a degree of chronic pulmonary hypertension. There was akinesis of the RV free wall (McConnell’s sign). Intra-cardiac masses had not been reported on the CT imaging.

Video 1 Colour Doppler across the patent foramen ovale. View Video 1 at http://movie-usa.glencoesoftware.com/video/10.1530/ERP-17-0027/video-1.
Download Video 1

Video 2 Intracardiac mass straddling the septal defect. View Video 1 at http://movie-usa.glencoesoftware.com/video/10.1530/ERP-17-0027/video-1.
Download Video 2

The case was discussed urgently with the on-call tertiary centre cardiologist and cardiac surgeon. The patient was intubated, ventilated and during transfer remained persistently hypotensive with a mean arterial blood pressure of 50–60 mmHg.

## Treatment and outcome

**Figure 3:**
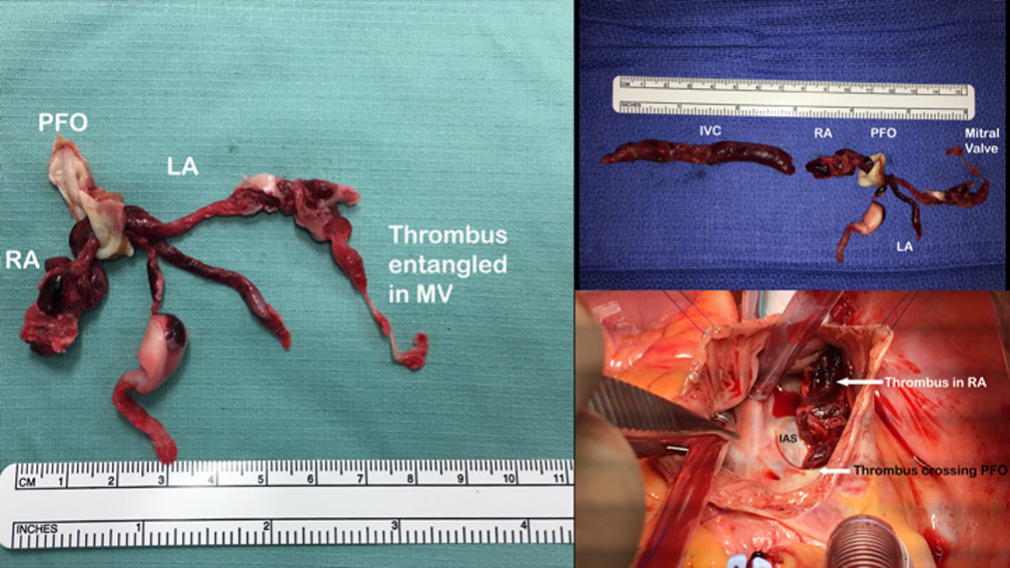
Intra-operative image demonstrating the burden of the thrombus network.

The patient was admitted to the acute medical unit with a plan to deliver thrombolytic therapy should the patient become more unstable overnight. The following morning, after the echocardiogram demonstrated significant intra-cardiac thrombus burden, it was deemed too high-risk to proceed with thrombolysis. Surgical removal of the thrombi and closure of the patent foramen ovale (PFO) was viewed as the most appropriate management option to prevent embolization with catastrophic consequences. Emergency surgery was undertaken that evening with a calculated Logistic EuroSCORE 20.95% given the critical preoperative state. The PFO and a wide margin of septum was excised in one piece accompanied by the large amount of organised thrombus arising from the inferior vena cava (Fig. 3), traversing the atrial septum and entangled in the sub-valvular mitral apparatus. The septum was patched using bovine pericardium and the patient was taken off cardiopulmonary bypass, but a small residual shunt was found on trans-oesophageal echo. This was of no haemodynamic significance, but taking into consideration the severity of the clinical presentation, prudently, this was deemed an inadequate result. The patient was re-established on cardiopulmonary bypass to fully close the septal defect, and on re-inserting the inferior vena caval cannula, a further 9 cm long, organised thrombus flushed out of the atrium by chance.

Recovery was complicated by transient atrial fibril­lation; however, the patient was discharged from Intensive Care at day 10, and to home on day 14 with a loop diuretic and oral anticoagulation, the latter to continue life-long. A battery of auto-antibodies including beta2 glycoprotein I, tissue transglutaminase and antiphospholipid antibodies were negative, immunoglobulins were normal, and no paraprotein bands were seen. Clotting, antithrombin activity and a lupus anticoagulant screen were normal. The masses were histologically confirmed as thrombus.

The patient’s ECG demonstrated persistent T wave inversion in leads V1–5 and pulmonary hypertension on post-operative echocardiogram consistent with chronic thromboembolic pulmonary hypertension (CTEPH). A dual-energy CTPA 12 months later arranged by the pulmonary hypertension service demonstrated resolution of the nodule in the lingula, right lower lobe infarct and right heart dilatation. Iodine perfusion demonstrated inhomogeneous iodine perfusion with no large segmental defects although there were multiple distal pulmonary artery occlusions and stenoses in keeping with CTEPH.

## Discussion

This case highlights a florid presentation of high-risk venous thromboembolism, and importantly, the treatment dilemmas in the setting of haemodynamic instability with large volume intra-cardiac thrombus burden. There are currently no guideline recommendations in this area. As mentioned in the ESC guidelines, the role of echocardiography in the patient with shock or hypotension is strengthened by the occasional finding of right heart thrombi (1) yet echocardiography is not mandated prior to primary reperfusion and, as in this case, intra-cardiac thrombi were not detected on CT. Thrombolysis has been used safely in the setting of intra-cardiac thrombus burden (2), however we felt that in a patient who is a surgical candidate, urgent cardiac surgery would be the safest approach to avoid shedding of the thrombus burden and embolization to vital organs with devastating consequences.

There is a mandate for echocardiography in all patients with pulmonary emboli, since certain features particularly guide management. Chronic pulmonary hypertension exists in 5–10% of patients (3) and patients with persistent features at discharge should be followed-up. Unprovoked venous thromboembolism may prompt screening for malignancy as in this case, where both a mildly raised PSA level (4.79) and lung nodule were followed up.

Lastly, the case highlights the masquerade and diversity in presentations of significant pulmonary emboli. Often inflammatory markers are significantly raised, yet a clear chest with a raised jugular venous pressure is a pertinent clue, and clinical acumen and suspicion are required to make a prompt diagnosis.

## Declaration of interest

The authors declare that there is no conflict of interest that could be perceived as prejudicing the impartiality of this case report.

## Funding

This work was supported by a Clinical Research Training Fellowship from the British Heart Foundation (Dr McConkey, FS/16/51/32365).

## Patient consent

The patient has willingly provided consent and a signed form has been provided.

## Author contribution statement

Dr McConkey and Professor Redwood wrote the article. Mr Ghosh-Dastidar and Professor Bapat were the attending surgeons and provided intra-operative images and article editing.

## References

[1] Konstantinides SV Torbicki A Agnelli G Danchin N Fitzmaurice D Galiè N Gibbs JS Huisman MV Humbert M Kucher N 2014 2014 ESC Guidelines on the diagnosis and management of acute pulmonary embolism. European Heart Journal 35 3033–3073. (10.1093/eurheartj/ehu283) 25173341

[2] Salman T Satija S Martin SF Sperling L 2010 Thrombolysis for saddle pulmonary embolism and 3-chamber thrombus. Texas Heart Institute Journal Texas Heart Institute 37 234–236. PMC2851412 20401303

[3] Lang IM Madani M 2014 Update on chronic thromboembolic pulmonary hypertension. Circulation 130 508–518. (10.1161/circulationaha.114.009309) 25092279

